# Supplementary material for: Antibacterial activity of the natural product elaiophylin against Streptococcus suis and its biofilm eradication effect
Source: Microbiol Spectr. 2026 Oct 8;14(11):e03949-25. doi: 10.1128/spectrum.03949-25 (PMC13729137; doi:10.1128/spectrum.03949-25)
Supplement: Supplementary file 1 [file spectrum.03949-25-s0001.docx]

Supplementary Materials

Fig. S1. LC-MS validation of compound 1[M + Na]^+^

Fig. S2. LC-MS validation of compound 2

Fig. S3. LC-MS validation of compound 3

Fig. S4. LC-MS validation of compound 4

Fig. S5. LC-MS validation of compound 5

Fig. S6. LC-MS validation of compound 6

Fig. S7. LC-MS validation of compound 7
